# Deciphering the code: the pivotal role of lncRNAs in advancing TNBC therapy

**DOI:** 10.3389/fonc.2024.1450980

**Published:** 2024-09-02

**Authors:** Weiping Chen, Zhiyong Pan, Zhengfu Feng, Xin Wang, Song Zhu

**Affiliations:** ^1^ Department of Respiratory, The Affiliated Qingyuan Hospital (Qingyuan People’s Hospital), Guangzhou Medical University, Qingyuan, China; ^2^ Department of Radiotherapy, The Affiliated Qingyuan Hospital (Qingyuan People’s Hospital), Guangzhou Medical University, Qingyuan, China

**Keywords:** triple-negative breast cancer (TNBC), long non-coding RNAs (lncRNAs), therapeutic targets, gene expression regulation, precision medicine

## Abstract

Triple-negative breast cancer (TNBC) represents the most formidable subtype of breast cancer, characterized by a notable dearth in targeted therapeutic options. Deciphering the underlying molecular mechanisms of TNBC is pivotal for improving patient outcomes. Recent scientific advancements have spotlighted long non-coding RNAs (lncRNAs) as key players in the genesis, progression, and metastasis of cancers. This review delineates the significant influence of lncRNAs on the advancement, detection, and neoadjuvant chemotherapy efficacy in TNBC, detailing the diverse expression patterns of aberrant lncRNAs. The paper explores the specific mechanisms by which lncRNAs regulate gene expression in both the nucleus and cytoplasm, with a special focus on their involvement in TNBC’s post-transcriptional landscape. Thorough investigations into TNBC-associated lncRNAs not only forge new avenues for early diagnosis and potent treatment strategies but also highlight these molecules as promising therapeutic targets, heralding an era of personalized and precision medicine in TNBC management.

## 1 Introduction

Triple-negative breast cancer (TNBC), as a distinct subtype of breast cancer, accounts for approximately 15% of all breast cancer cases (1). Its unique biological characteristics have made it a focal point of research in the field of breast cancer. TNBC is particularly notable because it lacks the expression of estrogen receptor (ER), progesterone receptor (PR), and human epidermal growth factor receptor-2 (HER2), rendering traditional hormone therapies and HER2-targeted therapies ineffective (2). TNBC exhibits aggressive biological behavior, characterized by rapid proliferation, high histological grade, and strong metastatic potential (3). This malignancy often leads to late-stage diagnosis, presenting significant treatment challenges and reducing patient survival expectations (4). Although some progress has been made in the treatment of TNBC in recent years, overall, the therapeutic options remain relatively limited. With a five-year survival rate of only 77%, TNBC is one of the leading threats to women’s health (5). To improve the prognosis of patients with TNBC, researchers continue to explore new treatment strategies and biomarkers.

Leveraging data from The Cancer Genome Atlas (TCGA), recent studies have revealed the molecular heterogeneity of TNBC through epigenetic analysis, identifying six molecular subtypes with unique biological characteristics and clinical prognoses (6). Among these, the basal-like BL1 subtype is characterized by high expression of Ki-67, NRAS, Myc, ATR, and BRCA genes; the BL2 subtype mainly exhibits high expression of MET, TP53, EGFR, and EPHA2 genes; the immunomodulatory subtype involves overexpression of genes related to immune cell signaling; the mesenchymal and mesenchymal stem-like subtypes are closely associated with chemoresistance; and the luminal androgen receptor (LAR) subtype is associated with better overall survival (OS) rates. The discovery of these subtypes provides a theoretical basis for the precision treatment of TNBC.

To enhance the survival rates of patients with TNBC, it is necessary to further identify biomarkers that can predict the risk of metastasis, treatment response, or even aid in the development of new therapies. In this field, research on long non-coding RNAs (lncRNAs) has emerged, showing that abnormal expression of lncRNAs is closely related to the development of TNBC (7–12). lncRNAs play important regulatory roles in eukaryotic cells, and their dysfunction is key in tumor formation. Research not only focuses on mutations or epigenetic modifications at the level of gene expression regulation but has also discovered that lncRNAs finely regulate post-transcriptionally, affecting mRNA stability and translation efficiency, thus regulating tumor cell growth, proliferation, and metastasis. The lncRNA expression profiles of tumors have become an important tool for distinguishing different types of cancer, providing new insights for the precise diagnosis and treatment of TNBC (**Table 1**).

**Table 1:** Important lncRNAs associated with triple-negative breast cancer.

LncRNAs	Expression	Biological function	Potential Targets	Reference
LINC00096	Up	Promote cell proliferation and invasion	miR‐383‐5p/RBM3	(19)
LUCAT1	Up	Enhances cellular proliferation, advances cell cycle progression, and promotes metastasis	miR‐5702	(20)
LINC01096	Up	Promote cell proliferation, migration, and invasion	miR‐3130‐3p	(21)
CCAT1	Up	Promote cell proliferation, migration, and invasion	miR‐218/ZFX	(22)
HOTAIR	Up	Promote cell proliferation, migration, and invasion;activating the Wnt/β-catenin signaling pathway	miR‐34a	(23–25)
MALAT1	Up	Promote cell proliferation, cell cycle arrest, and invasion	miR‐129‐5p; miR‐1/Slug; miR‐448/KDM5B	(26–28, 60)
DANCR	Up	Promote cell proliferation, migration, and invasion	miR‐216a‐5p; RXRA; CD44, ABCG2	(32–34)
H19	Up	Bolster tumor cell proliferation and survival	Akt	(41–44)
GAS5	Down	Inhibit cell proliferation and invasion; Promote cell apoptosis	miR‐378a‐5p, miR‐196a‐5p	(47–50)
PVT1	Up	Promote cell proliferation and migration;aerobic glycolysis	miR-145-5p; miR-497	(52–55)

## 2 The biological functions of lncRNAs

LncRNAs are a unique class of RNA molecules exceeding 200 nucleotides in length, lacking the capacity to encode proteins (13). They exhibit distinctive expression patterns in specific stages of tissue differentiation or certain types of cancer, making them key molecules in the study of cellular biology and disease mechanisms.

LncRNAs reside in the nucleus or cytoplasm (14), overlapping with coding and non-coding RNA transcripts. Based on their genomic proximity to adjacent genes, lncRNAs are categorized into five main types (15): sense lncRNAs, overlapping with one or more exons of a protein-coding gene on the same strand; antisense lncRNAs, overlapping with one or more exons of a protein-coding gene on the opposite strand; bidirectional lncRNAs, initiating expression less than 1000 base pairs away from a neighboring coding transcript on the opposite strand; intronic lncRNAs, originating from an intron of another transcript; and intergenic lncRNAs, existing independently within the genomic interval between two genes.

In the realm of pathology, particularly within the scope of cancer diagnostics, the exploration of lncRNAs holds paramount significance. These molecular entities are not mere harbingers of prognostic insights but also pivotal in steering therapeutic decisions. As sentinels of cellular dynamics and architects of gene regulatory frameworks, lncRNAs orchestrate gene expression through a spectrum of modalities, encompassing chromatin reconfiguration, transcriptional governance, and post-transcriptional modulation (16).

Empirical evidence underscores the integral role of lncRNAs in modulating a myriad of biological functions, including cellular proliferation, programmed cell death, cell cycle regulation, tissue invasion, metastatic spread, cellular differentiation, chromatin architecture, and intracellular transport mechanisms (17, 18). Notably, the unique expression patterns of lncRNAs in specific types of cancer highlight their potential as diagnostic markers. For instance, the upregulation of certain lncRNAs in breast cancer has been linked to tumor aggressiveness and poor prognosis, making them valuable for early detection and risk stratification.

Furthermore, the ability of lncRNAs to regulate gene expression at multiple levels positions them as promising therapeutic targets. Targeting specific lncRNAs could disrupt cancer-promoting pathways, thereby offering new avenues for cancer treatment. For example, inhibiting the expression of lncRNAs that promote angiogenesis in glioblastoma could potentially slow tumor growth and improve patient outcomes.

Consequently, profound investigations into lncRNAs transcend the mere elucidation of disease pathogenesis. They unveil novel diagnostic markers and therapeutic targets, heralding innovative trajectories in the management and treatment of malignancies. The discovery of lncRNAs as potential biomarkers and therapeutic targets could revolutionize personalized medicine, enabling more precise and effective treatment strategies tailored to individual patient needs.

## 3 Roles of lncRNAs in TNBC

### 3.1 Regulating the growth of breast cancer cells

In the intricate landscape of TNBC, lncRNAs are emerging as pivotal elements. LINC00096 is known to markedly enhance TNBC cell proliferation, likely through its interplay with miR-383-5p and the modulation of RBM3, an RNA-binding protein (19). While the direct effects of LINC00096 on the cell cycle are yet to be fully elucidated, its influence on cell proliferation intimates a potential indirect role in cell cycle dynamics.

LUCAT1 (lung cancer associated transcript 1) propels the division and growth of TNBC cells by orchestrating gene expression and cell cycle processes via miR-5702 (20). Its elevated expression levels enable tumor cells to circumvent programmed cell death, thereby fostering tumor expansion and contributing to invasion and metastasis. Thus, LUCAT1 stands as a critical factor in TNBC progression, offering itself as a promising therapeutic target and a significant prognostic marker.

The distinct lncRNA LINC01096 is implicated in the proliferation, migration, and invasion of TNBC cells, while concurrently impeding apoptosis, potentially through the regulatory pathways involving miR-3130-3p (21). The expression of LINC01096 bears a strong correlation with TNBC patients’ resistance to certain treatments, hinting at a possible association with chemotherapy resistance. Consequently, LINC01096 serves as a valuable biomarker for diagnosis, prognosis, and treatment responsiveness, as well as a prospective avenue for novel therapeutic approaches.

CCAT1 (Colon Cancer Associated Transcript 1) assumes a notably critical role in TNBC, with its expression intricately linked to the migratory and invasive traits of the cells (22). By modulating the epithelial-mesenchymal transition (EMT) via the miR-218/ZFX signaling axis, CCAT1 may amplify tumor virulence and the propensity for metastasis (22). The expression levels of CCAT1 are also tied to treatment resistance in TNBC patients, positioning it as a potential biomarker for disease diagnosis, prognostic evaluation, or treatment response, and as a target for emerging therapeutic strategies.

### 3.2 Regulating the invasion and metastasis of breast cancer cells

In the intricate tapestry of TNBC, specific lncRNAs such as HOTAIR and MALAT1 are emerging as critical players. HOTAIR is intricately linked to TNBC’s invasiveness and metastatic potential, orchestrating chromatin remodeling and activating the Wnt/β-catenin signaling pathway (23, 24). Its elevated expression correlates with a grim prognosis, marked by advanced tumor grade, lymph node metastasis, and distant spread. Furthermore, HOTAIR modulates the epithelial-mesenchymal transition (EMT) through the miRNA sponge mechanism, a pivotal process for tumor invasion and metastasis (25). It is also implicated in therapeutic resistance in TNBC, potentially inducing chemotherapy drug resistance by altering drug metabolism and cell apoptosis pathways. Thus, HOTAIR serves as a potential biomarker for TNBC diagnosis, prognosis assessment, and treatment response monitoring, as well as a strategic target for novel therapeutic interventions.

Similarly, MALAT1’s increased expression augments tumor cell migration and invasion, engaging key signaling pathways like PI3K/AKT/mTOR and Wnt/β-catenin (26, 27). MALAT1 also facilitates TNBC progression by modulating intercellular interactions within the tumor microenvironment. Its strong association with TNBC’s invasiveness and metastatic potential positions MALAT1 as a potential prognostic biomarker. At the molecular level, MALAT1 functions as a ‘sponge’ for miRNAs, such as miR-129-5p, influencing miRNA-mediated gene regulation (28).

Other lncRNAs, including MIR503HG, sONE, and ZEB2-AS1, also significantly contribute to TNBC’s pathogenesis. MIR503HG effectively curtails TNBC cell migration and invasion via the miR-103/OLFM4 axis (29). sONE hampers these processes by affecting signaling pathways triggered by eNOS and NO production (30). ZEB2-AS1 is suspected of promoting EMT through the PI3K/Akt/GSK3β/ZEB2 signaling cascade (31). The expression levels of these lncRNAs are intimately connected to TNBC’s invasiveness and metastatic capacity, highlighting their potential as biomarkers for diagnosis and treatment response.

The lncRNA DANCR is associated with heightened proliferation and invasion of tumor cells, potentially through its influence on specific signaling pathways or miRNA interactions, such as with miR-216a-5p (32). DANCR also engages the PI3K/Akt pathway by binding to the retinoid X receptor alpha (RXRA), a key player in TNBC cell proliferation and invasion (33). Moreover, DANCR fosters TNBC stem cell-like traits by regulating tumor stem cell marker expression, including CD44 and ABCG2 (34). Its upregulation is also linked to chemotherapy resistance, possibly due to its involvement in cell cycle and apoptosis regulation. Therefore, DANCR is not merely a potential biomarker for TNBC diagnosis and treatment monitoring but also a vital target for innovative therapeutic strategies.

### 3.3 Regulating the tumor microenvironment

Within the tumor microenvironment, select lncRNAs assume a critical role, engaging in dynamic interactions with cellular components, the extracellular matrix, immune cells, and vasculature. These interactions are instrumental in modulating tumor growth, tissue invasion, metastatic spread, and resistance to therapies. Gaining a comprehensive understanding of the operational mechanisms of these lncRNAs is essential for the innovation of novel therapeutic approaches. Their study not only illuminates the complex interplay within the tumor milieu but also provides a foundation for targeted treatment strategies that could revolutionize cancer care.

NEAT1 (nuclear paraspeckle assembly transcript 1), a lncRNA distinguished by its nuclear enrichment, has emerged as a focal point of interest due to its multifaceted influence within the tumor microenvironment. Research indicates that NEAT1 modulates tumor evolution through a plethora of mechanisms (35–40): it orchestrates immunomodulation by impacting immune cells like T cells, dendritic cells, and NK cells, thereby fine-tuning anti-tumor immune responses; it fosters interactions between tumor and stromal cells, intensifying invasion and metastasis; it contributes to angiogenesis by guiding the proliferation and migration of endothelial cells, facilitating the development of the tumor’s vascular network; it remodels the extracellular matrix (ECM), reshaping the tumor microenvironment’s physical attributes; it partakes in metabolic reprogramming, influencing the metabolic circuits of both tumor and stromal cells; it governs pivotal signaling pathways, including PI3K/Akt and MAPK/ERK, essential for tumor cell proliferation, survival, and invasion; it modulates cancer stem cell traits, impacting tumor recurrence and drug resistance; it is implicated in chemotherapy resistance, potentially through the modulation of gene expression linked to drug metabolism and apoptosis; it advances EMT, bolstering the invasive and metastatic capacities of tumor cells; and it acts as a competitive endogenous RNA (ceRNA), regulating miRNA activity, thus altering gene expression within the tumor microenvironment. NEAT1’s actions weave a complex regulatory web, pivotal to the tumor’s ecological dynamics.

H19, an lncRNA aberrantly expressed in various malignancies, notably TNBC, orchestrates a complex array of mechanisms within the tumor microenvironment. It modulates pivotal signaling pathways, including PI3K/Akt, to bolster tumor cell proliferation and survival (41). H19 is intimately linked to cancer stem cell traits, potentially influencing self-renewal and differentiation through gene expression regulation (42). It also shapes the behavior of immune cells, affecting their infiltration, activation, and function, thereby contributing to tumor immune evasion (43). In the realm of angiogenesis, H19 facilitates the proliferation and migration of endothelial cells, supplying the tumor with essential oxygen and nutrients (44). It governs genes implicated in cell invasion and metastasis, such as those involved in the EMT process, enhancing the invasive and metastatic potential of tumor cells. The expression level of H19 is associated with tumor cell resistance to chemotherapy, possibly by modulating genes related to drug metabolism and apoptosis (41). Furthermore, H19 partakes in the remodeling of the ECM, altering the tumor microenvironment’s physical landscape to favor tumor cell invasion and metastasis (45). As a ceRNA, it regulates miRNA activity, thereby influencing gene expression within the tumor milieu. H19 may also interact with other non-coding RNAs, proteins, or metabolites, maintaining the homeostasis of the tumor microenvironment (18, 46). Consequently, the expression level of H19 serves as a biomarker for cancer diagnosis, prognosis evaluation, and monitoring treatment response in the tumor microenvironment.

GAS5 (growth arrest specific 5), a lncRNA, has garnered significant attention in oncology, especially in the context of TNBC. Within the tumor microenvironment, GAS5 exerts multifaceted roles: it curtails tumor cell proliferation and fosters apoptosis, mediated by interactions with miRNAs or modulation of genes governing the cell cycle and apoptosis. GAS5 engages with specific miRNAs, such as miR-196a-5p and miR-378a-5p, to influence the expression of genes related to the cell cycle, thereby impacting tumor cell proliferation (47, 48). It plays a role in enhancing chemosensitivity, with its expression levels correlating with tumor cell responsiveness to chemotherapy agents, potentially heightening their susceptibility by regulating genes linked to drug resistance (49). In the sphere of immunoregulation, GAS5 influences immune-related genes or miRNAs, thereby affecting immune cells like T cells and NK cells, which is crucial for modulating tumor immune evasion. It also impedes tumor angiogenesis by altering the expression of angiogenesis-related genes, thus inhibiting the formation of the tumor vascular network (50). GAS5 regulates genes associated with tumor metastasis, including those involved in the EMT process, influencing the invasiveness and metastatic capabilities of tumor cells (51). Functioning as a ceRNA, it partakes in the regulation of miRNA activity, affecting their regulatory impact on target genes via miRNA interactions (50). The expression level of GAS5 serves as a biomarker for cancer diagnosis and prognosis, mirroring the biological attributes of the tumor and patient clinical outcomes (50). Additionally, GAS5 is implicated in reversing chemotherapy resistance by modulating specific signaling pathways or miRNAs and plays a role in regulating metabolic processes within the tumor microenvironment, affecting the energy metabolism and biosynthesis of tumor cells (50).

### 3.4 Regulating aerobic glycolysis in tumor cells

Certain lncRNAs exert a profound influence on the energy metabolism of TNBC cells. They achieve this objective through a variety of mechanisms, particularly by directly or indirectly regulating key enzymes and transport proteins within the glycolytic pathway. An in-depth exploration of these lncRNAs’ roles in energy metabolism not only sheds light on the adaptive metabolic strategies of TNBC cells but also holds the potential to unlock novel avenues for therapeutic intervention in cancer treatment.

PVT1, a long non-coding RNA frequently upregulated in diverse cancers, is intimately linked with the proliferative, invasive, and metastatic propensities of tumor cells. Within the context of aerobic glycolysis, known as the Warburg effect, PVT1 assumes a critical role, potentially orchestrating the expression and functionality of essential enzymes in the glycolytic pathway to fulfill the heightened energy demands of rapidly dividing tumor cells (52). Moreover, PVT1 modulates the metabolic landscape of tumor cells by adjusting key metabolites in the tumor microenvironment, such as lactate and pyruvate, which in turn serve as signaling entities influencing tumor cell conduct (53). Functioning as a ceRNA, PVT1 engages with miRNAs, alleviating the repression of crucial glycolytic genes, thus fostering aerobic glycolysis (54). It may further amplify this process via interactions with the HIF-1α (Hypoxia-Inducible Factor-1α) signaling pathway, a pivotal regulator of glycolytic gene expression (55). Most significantly, PVT1’s impact on metabolic reprogramming entails a reconfiguration of cellular metabolic routes, bolstering tumor growth and viability (54). This metabolic flexibility enables tumor cells to endure metabolic stress, with PVT1 playing an indispensable role in this adaptive survival strategy.

UCA1 (Urothelial Carcinoma Associated 1), a lncRNA aberrantly expressed in a spectrum of cancers, is intricately associated with the proliferative, invasive, metastatic, and metabolic attributes of tumor cells. UCA1 assumes a multifaceted role in glycolysis, potentially steering the expression or functionality of critical enzymes, thereby directly influencing tumor cell glycolysis (56). Concurrently, it modifies metabolites within the tumor microenvironment, impacting both glycolysis and the metabolic state of tumor cells (57). As a ceRNA, UCA1 engages with miRNAs, alleviating the repression of essential glycolytic genes, thus further propelling glycolysis (58). It may also indirectly foster glycolysis via interactions with the HIF-1α signaling pathway (59). By dictating the metabolic reprogramming of tumor cells and reshaping cellular metabolic pathways, UCA1 underpins tumor growth and sustenance (56). This metabolic versatility permits tumor cells to withstand metabolic stress, with UCA1 playing a pivotal role. Ultimately, by catalyzing glycolysis, UCA1 supplies the requisite energy and biosynthetic precursors for tumor cells, bolstering their invasive and metastatic prowess.

### 3.5 Regulating the apoptosis of breast cancer cells

LncRNAs are crucial in regulating apoptosis, a vital process for maintaining tissue homeostasis and preventing tumor development. In the context of TNBC, specific lncRNAs influence apoptosis through various mechanisms, significantly impacting tumor progression and metastasis.

MALAT1 has been identified as a key player in modulating apoptosis in TNBC. It is suggested that MALAT1 can regulate apoptosis by interacting with miRNAs, such as miR-1 (60). Research indicates that Slug, a transcription factor essential for cell migration, invasion, and apoptosis, is a direct target of miR-1 and may be regulated by MALAT1. Downregulation of MALAT1 leads to an increase in miR-1 expression, which can promote apoptosis by targeting specific mRNAs. By suppressing apoptosis, MALAT1 potentially enhances the invasiveness and metastatic potential of tumor cells, increasing the malignancy of the tumor.

Furthermore, NEAT1, another nuclear-enriched lncRNA, is implicated in TNBC development through its influence on apoptosis-related signaling pathways. NEAT1 may regulate the expression of proteins that either inhibit or promote apoptosis via interactions with miRNAs (40). Additionally, it may indirectly preserve genomic stability by affecting DNA damage response and repair mechanisms, thus impacting apoptosis. As a ceRNA, NEAT1 can modulate the expression of miRNA target genes involved in apoptosis regulation, such as cyclin E1 and cyclin D1 (40).

The intricate and multifaceted regulation of apoptosis by lncRNAs in TNBC underscores the complexity of the disease’s molecular landscape. By dissecting the interactions between these lncRNAs, miRNAs, and their target genes, we can gain a deeper understanding of TNBC’s molecular underpinnings. This knowledge is invaluable for identifying potential therapeutic targets and developing novel strategies to combat this aggressive form of cancer.

### 3.6 Regulating the angiogenesis of breast cancer cells

Angiogenesis, the intricate process of new blood vessel formation, is a pivotal mechanism underpinning the growth and metastatic cascade of breast cancer. The orchestration of angiogenesis within breast cancer cells results from a complex interplay among numerous regulatory factors. Within this landscape, lncRNAs have emerged as key modulators with significant implications for cancer progression.

HOTAIR, an lncRNA exhibiting aberrant expression across various cancers, has been particularly implicated in TNBC due to its high expression levels. These levels correlate closely with the tumor’s invasive edge, metastatic propensity, and angiogenic activity. HOTAIR’s influence on angiogenesis is multifaceted; it is postulated to reshape the tumor microenvironment by orchestrating the delicate balance of cytokines and growth factors, including VEGF and FGF (61). This modulation paves the way for new blood vessel formation, thereby nurturing a conducive milieu for tumor cells and amplifying their invasive and metastatic prowess. Moreover, HOTAIR’s reach extends to the immunological realm of the tumor, potentially skewing the polarization of tumor-associated macrophages (TAMs) and implicating itself in the dual narratives of angiogenesis and immune evasion within the tumor (61).

ANRIL, another lncRNA with disrupted expression in various cancers, mirrors HOTAIR’s impact on TNBC’s angiogenesis. Current understanding suggests that ANRIL’s role is mediated through the regulation of genes and signaling pathways pivotal to angiogenesis, such as the VEGF and Notch pathways (62). As a ceRNA, ANRIL adds layers of complexity to its regulatory influence by acting as a sponge for specific miRNAs, thereby modulating the expression of a spectrum of target genes involved in angiogenic processes (63). Its ripple effects on the tumor microenvironment, touching upon immune and stromal cells, further highlight ANRIL’s therapeutic potential in TNBC.

The discovery of lncRNAs like HOTAIR and ANRIL as conductors of angiogenesis unveils fresh avenues for targeted therapeutics in TNBC. Targeting these lncRNAs to inhibit their function or expression may critically disrupt the angiogenic machinery of tumors, effectively starving them of the vital blood supply imperative for growth and metastasis. As we stand on the precipice of new discoveries, future research must diligently unravel the intricate mechanisms through which these lncRNAs operate, identify their synergistic or antagonistic interplays with other regulatory molecules, and rigorously assess the safety and efficacy of lncRNA-targeted therapies in the preclinical and clinical spectrum. This pursuit will not only deepen our comprehension of the molecular underpinnings of TNBC but also illuminate the path toward innovative and efficacious treatment modalities.

### 3.7 Regulating the stemness of breast cancer cells

Cancer stem cells (CSCs) possess remarkable abilities of self-renewal and multi-lineage differentiation, which are pivotal in the genesis, progression, metastasis, and resistance to therapy of tumors. Their stem-like characteristics enable these cells to foster the recurrence and relentless growth of tumors, complicating the landscape of cancer treatment. In breast cancer, especially the aggressive TNBC, the presence of CSCs is identified as a principal factor contributing to a grim prognosis. Thus, deciphering and targeting the molecular underpinnings that govern the properties of CSCs is essential for the formulation of efficacious therapeutic approaches.

In TNBC, lincRNA-ROR emerges as a pivotal lncRNA in modulating the characteristics of cancer stem cells. Evidence indicates that lincRNA-ROR is markedly overexpressed in TNBC tissues, in stark contrast to non-cancerous tissues, and it facilitates the initiation and spread of cancer by orchestrating the EMT (64). The suppression of lincRNA-ROR is observed to curb the EMT phenotype in TNBC cells. Advanced deep sequencing has unveiled that the diminished expression of miR-145 is indicative of metastatic potential, a process intricately linked to regulation by lincRNA-ROR (65). lincRNA-ROR is hypothesized to function as a ceRNA to miR-145, thereby constraining miR-145 levels in TNBC. Moreover, both miR-145 and lincRNA-ROR are implicated in diverse stages of embryonic and adult stem cell development. lincRNA-ROR is shown to modulate the expression of a suite of stemness factors, such as SOX2, OCT4, and NANOG, by sequestering miR-145 (66).

NEAT1, another lncRNA significantly overexpressed in TNBC, assumes a critical role in the regulation of tumor stemness. NEAT1 propels the advancement of cancer by spurring cell proliferation, EMT, invasion, and metastasis, and it instigates resistance to chemotherapy in TNBC cells. At the molecular level, NEAT1 is found to foster breast cancer growth by modulating miRNAs, including miR-548, miR-448, and ZEB1 (40, 67). The overabundance of NEAT1 is linked to the suppression of miR-448 expression, thereby liberating ZEB1 to manifest its influence. The inhibition of NEAT1 has been shown to deplete the reservoir of cancer stem cells endowed with robust self-renewal and multi-lineage differentiation capabilities, underscoring NEAT1’s cardinal role in the regulation of tumor stemness.

lncRNAs such as lincRNA-ROR and NEAT1 play indispensable roles in the modulation of TNBC stem cell properties. Through a tapestry of intricate molecular mechanisms—ranging from acting as ceRNAs to influence miRNA expression to impacting the functionality of stemness factors, either directly or indirectly—they synergize to propel the malignant evolution of TNBC. These revelations not only amplify our comprehension of the regulatory dynamics of TNBC cancer stem cells but also chart new horizons and potential therapeutic targets for the development of precision medicine aimed at cancer stem cells.

### 3.8 Regulating the drug resistance of breast cancer cells

The challenge of drug resistance in TNBC significantly undermines the effectiveness of chemotherapy. The emergence of resistance mechanisms frequently culminates in treatment failure and disease recurrence. LncRNAs have emerged as pivotal regulators in the intricate dynamics of drug resistance, with their misregulation being linked to altered chemosensitivity in TNBC cells.

The lncRNA FTH1P3 epitomizes the complex interplay between lncRNAs and drug resistance. Research has illuminated its role in modulating paclitaxel resistance in TNBC via the miR-206/ABCB1 axis, with ABCB1 being a quintessential chemotherapy resistance protein found in multidrug-resistant cancers (68). In paclitaxel-resistant cells, such as the MDA-MB-231/PTX line, the overexpression of lncRNA FTH1P3 and its subsequent suppression have been shown to enhance paclitaxel sensitivity by 50%, underscoring the lncRNA’s pivotal role in chemotherapy resistance mechanisms. Moreover, *in vivo* studies using transplants of paclitaxel-resistant cells have demonstrated that silencing FTH1P3 leads to diminished ABCB1 protein levels and curtailed tumor growth, highlighting its direct influence on the miR-206/ABCB1 signaling pathway.

Similarly, the lncRNA H19 has been recognized as an overexpressed entity in breast cancer that confers resistance to chemotherapy (41). Its elevated expression in paclitaxel-resistant TNBC cells and the reversion to chemosensitivity following H19 knockdown underscore its contribution to the resistant phenotype (41). The Akt signaling pathway, often dysregulated in breast cancer, is implicated in treatment resistance. Knockdown of H19 initiates apoptosis mediated by the Akt pathway, thereby sensitizing TNBC cells to paclitaxel (41). Clinical trials corroborate the relevance of targeting the Akt pathway, as the combination of Ipatasertib with paclitaxel has been shown to markedly enhance progression-free survival in patients with metastatic TNBC (69).

Investigating lncRNAs such as FTH1P3 and H19 in the context of TNBC chemotherapy resistance offers profound insights into the molecular underpinnings of drug resistance. These lncRNAs not only serve as biomarkers for chemotherapy resistance but also represent promising therapeutic targets. By modulating the expression of proteins critical to drug efflux and apoptosis pathways, these lncRNAs offer a means to devise strategies aimed at overcoming TNBC chemotherapy resistance. Future research should focus on delineating the comprehensive regulatory networks of these lncRNAs and identifying effective combination therapies that can target these lncRNAs, thereby augmenting TNBC’s responsiveness to chemotherapy.

## 4 lncRNAs as potential therapeutic targets for TNBC treatment

In the realm of TNBC research, lncRNAs have risen to prominence for their pivotal role in cellular biology and oncogenesis (70, 71). The dysregulation of lncRNAs across a spectrum of cancers underscores the potential for lncRNA-centric therapeutic strategies. Contemporary studies have enriched our comprehension of lncRNA functions in TNBC, especially concerning their influence on drug resistance and cancer progression. The pursuit of lncRNA-targeted drugs stands at the forefront of oncological innovation, heralding a new era in cancer therapeutics. Given the aberrant expression of lncRNAs in malignancies and their regulatory capacity over tumor growth, invasion, metastasis, and treatment resistance, they are increasingly recognized as auspicious targets for therapeutic intervention.

Antisense oligonucleotides (ASOs) are synthetic nucleic acid polymers designed to bind specifically to complementary sequences of lncRNAs, effectively silencing their activity (72). The strategic deployment of ASOs against targeted lncRNAs can substantially diminish their presence in tumor cells, thereby modulating cellular behavior. ASOs act as precision molecular instruments, influencing RNA processing and subsequent protein synthesis through a variety of pathways. Advances in ASO architecture and chemical refinement have markedly amplified their therapeutic promise and efficacy (73). A multitude of ASOs are currently advancing through clinical trials, showcasing the breadth and ingenuity of ASO-driven therapeutic modalities (74). In the context of TNBC, ASOs have been employed to downregulate lncRNA targets implicated in oncogenesis. For instance, the lncRNA TROJAN, known to facilitate TNBC proliferation and metastasis, correlates with diminished patient survival (75). Treatment with TROJAN-targeted ASOs has led to a notable decrease in metastatic lung nodules in murine models, following direct transfection into TNBC cells and systemic administration in xenograft scenarios, all while maintaining a minimal toxicity profile (75). These observations herald the potential of ASO-mediated TROJAN regulation as an innovative therapeutic avenue for TNBC. Additionally, ASOs aimed at lncRNA NRAD1 have demonstrated an ability to decelerate tumor progression and suppress TNBC stem cell-like properties (76). Research also reveals that targeting lncRNA MALAT1 with ASOs can lower its RNA abundance, culminating in retarded TNBC growth, diminished cellular proliferation, and heightened apoptosis (77). Experimental models have further indicated that MALAT1 suppression reshapes the tumor microenvironment, characterized by a decrease in immunosuppressive elements such as tumor-associated macrophages (TAMs) and myeloid-derived suppressor cells (MDSCs), coupled with an upsurge in cytotoxic CD8+T cells (77). In preclinical settings, the MALAT1-targeting ASO, when combined with chemotherapy or immune checkpoint blockade (ICB), has shown promise in augmenting therapeutic outcomes (77).

RNA nanotechnology plays a crucial role in the study of lncRNAs. This technology utilizes nanoscale tools and methods to delve into the role of lncRNAs in cellular functions and genomic stability (78). With RNA nanotechnology, scientists can more precisely reveal the structure and function of lncRNAs and study their impact on gene expression and disease formation (78). By designing nanoparticles that can specifically target and regulate the activity of lncRNAs, RNA nanotechnology not only can control gene expression but also achieve refined gene regulation (78). Additionally, RNA nanoparticles can serve as drug delivery carriers, transporting therapeutic molecules directly to specific cells or tissues. In the treatment of TNBC, the application prospects of RNA nanotechnology are particularly broad. For example, the expression level of lncRNA DANCR is abnormally elevated in TNBC and closely related to tumor development (34). Scientists have successfully developed a new type of nanoparticle—RGD-PEG-ECO/siDANCR, which is self-assembled from the multifunctional amino lipid ECO, cyclic RGD peptide-PEG, and siDANCR, and is specifically designed for systemic delivery to TNBC (79). In MDA-MB-231 and BT549 cell lines, after treatment with RGD-PEG-ECO/siDANCR nanoparticles, the expression of DANCR decreased by 80-90% within 7 days, demonstrating efficient siRNA delivery capability and sustained gene silencing effect (79). Moreover, these nanoparticles exhibited outstanding therapeutic effects *in vitro*, significantly reducing the invasion, migration, survival, spheroid formation, and proliferation capabilities of TNBC cells. At the molecular level, the effective inhibition of DANCR dynamically regulated the oncogenic network, by reducing the levels of PRC2-mediated H3K27me3 and inhibiting the Wnt/EMT signaling pathway, while also altering the phosphorylation patterns of various kinases in TNBC cells (79). In animal models, systemic administration of RGD-PEG-ECO/siDANCR nanoparticles at a siRNA dose of 1 mg/kg to nude mice with TNBC xenografts showed significant inhibitory effects on TNBC progression, without observable severe toxic side effects, highlighting the therapeutic potential and safety of these nanoparticles (79). Additionally, LINC00511, as a target gene related to drug resistance, has also attracted attention for its potential role in TNBC treatment. Researchers have constructed a new type of theranostic agent—a nanobubble complex carrying LINC00511-siRNA, for siRNA delivery, and evaluated its impact on drug sensitivity in TNBC models (80). The study results indicate that the nanobubble complex is an efficient and safe siRNA transfection tool. These findings provide a new perspective for RNA nanoparticle-based targeted treatment strategies, with the potential to bring breakthrough progress in the treatment of TNBC and other cancers through specific targeting and inhibition of lncRNAs.

The therapeutic promise of lncRNA-based strategies, including ASOs and RNA nanotechnology, is considerable. However, several hurdles must be surmounted to achieve their full clinical potential. A paramount challenge is the refinement of delivery systems. Ensuring the precise delivery of ASOs and RNA nanoparticles to tumor sites, while circumventing off-target effects and minimizing toxicity, is essential. The quest for targeted delivery systems that leverage tumor-homing peptides or specific ligands is a vibrant field of research (81). Moreover, the biocompatibility and biodegradability of these systems are crucial for ensuring patient safety.

Identifying the most efficacious lncRNA targets is a sophisticated endeavor, given the multitude of lncRNAs and their intricate roles in TNBC. A rigorous vetting process involving bioinformatics, experimental studies, and clinical data is imperative to guarantee the specificity and potency of therapeutic interventions. The ongoing quest to optimize ASO and nanoparticle design to bolster binding affinity, stability, and cellular uptake is a testament to the dynamic nature of this field (77, 82). The journey from bench to bedside is not without its obstacles, including the scalability of production, navigating regulatory landscapes, and the considerable financial investment required for personalized medicine. Tackling these issues necessitates a synergistic approach, with researchers, clinicians, and industry stakeholders collaborating to forge standardized protocols and economically viable manufacturing processes.

Looking ahead, future research endeavors should concentrate on deepening our comprehension of the molecular underpinnings of lncRNAs in TNBC pathogenesis, with a focus on the intricate interplay between lncRNAs and other cellular constituents, such as proteins and miRNAs, which may modulate treatment efficacy. The exploration of synergistic combination therapies that marry lncRNA-targeting approaches with traditional treatments like chemotherapy, radiotherapy, and immunotherapy could amplify therapeutic impact and potentially surmount resistance. The advancement of personalized medicine, facilitated by the discovery of predictive biomarkers for patient stratification and treatment response monitoring, will enable the tailoring of lncRNA-targeted therapies to individual patient profiles, enhancing the likelihood of favorable outcomes.

lncRNA-targeted therapeutics for TNBC, encompassing ASOs and RNA nanotechnology, stand at the vanguard of oncological innovation. Nonetheless, the route to clinical application is replete with challenges that demand persistent investigation and advancement. By surmounting current limitations, honing delivery systems, pinpointing and refining therapeutic targets, and charting a course for future research, we can illuminate a path toward treatments that are safer, more efficacious, and exquisitely tailored to the unique needs of TNBC patients.

## 5 Conclusion

In summary, lncRNAs play a multifaceted role in the progression and treatment of TNBC, ranging from modulating tumor cell proliferation and metastasis to serving as potential therapeutic targets. As our understanding of lncRNA functions and regulatory mechanisms deepens, lncRNA-based therapeutic strategies are emerging at the forefront of TNBC treatment. The advancement of technologies such as antisense oligonucleotides, locked nucleic acids, and RNA nanoparticles opens up new possibilities for the diagnosis and treatment of TNBC. These modalities have demonstrated potential not only *in vitro* but also in animal models, showcasing their safety and efficacy. Looking ahead, these studies hold the promise of delivering more personalized and precise treatment options for patients with TNBC.
